# Identification of hearing loss relevant genes in QTL on mouse chromosome 16

**DOI:** 10.1186/1471-2105-13-S12-A8

**Published:** 2012-07-31

**Authors:** Fusheng Zhao, Yonghui Ma, Yan Jiao, Lishi Wang, Yue Huang, Wei Wei, Weikuan Gu

**Affiliations:** 1 Department of Orthopedic Surgery-Campbell Clinic and Pathology, University of Tennessee Health Science Center, Memphis, TN 38163, USA; 2 Department of Histology and Embryology, Mudanjiang Medical College, Mudanjiang, PR China

## Background

Previously, a quantitative trait loci (QTL) for hearing loss on chromosome 16 in a 118 TNE and CAST background has been identified. The overlap of two of interval specific congenic recombinant strains (ISCRS) strains reduced the QTL interval into a 5,378,407 bps genome region.

## Results

By examining the genomic information of the QTL on chromosome 16, between the two flanking markers, D16mit191 and D16mit86, we identified a total of 84 genetic elements in the 5,378,407 bps genome region. Among these genetic elements, we found seven with potential function in hearing loss preference (Table 1). We then examined the SNPs, insertions and deletions, and gene expression levels of those seven genes.

**Table 1:** Candidate genes for hearing loss on Chr 16

Ensembl Accession	Symbol	Full Name
ENSMUSG00000039639	Kcne1	potassium voltage-gated channel
ENSMUSG00000022949	CLIC6	Chloride intracellular channel 6
ENSMUSG00000022952	RUNX1	Runt-related transcription factor 1
ENSMUSG00000079514	SLC5A3	Sodium/myo-inositol cotransporter
ENSMUSG00000022982	Sod1	superoxide dismutase 1
ENSMUST00000047383	Kcne2	potassium voltage-gated channel
ENSMUSG00000022967	Ifnar1	interferon (alpha and beta) receptor 1

## Conclusions

Our current data suggest that the Kcne1 and Sod1 genes are potentially the most hearing loss relevant genes. However, further experiments and examination are still needed to confirm their candidacy. Several other candidate genes are also in the process of being identified.

